# Editorial: Decision making from the perspective of neural thermodynamics and molecular information processing

**DOI:** 10.3389/fnins.2022.910996

**Published:** 2022-08-16

**Authors:** Reza Rastmanesh, Eva Deli, Sisir Roy, Brent Vogt

**Affiliations:** ^1^American Physical Society, College Park, MD, United States; ^2^Department of Anatomy, Histology and Embryology, University of Debrecen, Debrecen, Hungary; ^3^National Institute of Advanced Studies, Bangalore, India; ^4^Department of Anatomy and Neurobiology, Cingulum Neurosciences Institute, Manlius, NY, United States; ^5^Department of Anatomy and Neurobiology, School of Medicine, Boston University, Boston, MA, United States

**Keywords:** decision making, thermodynamics, information processing, neural circuit, molecular mechanism

Thermodynamics studies the relations between heat, work, temperature, and energy. The laws of thermodynamics describe how the energy in a system changes and whether the system can perform useful work on its surroundings. Thermodynamics has proved fruitful in many fields, such as chemistry, biology, and economics, and gives us hope for cognitive analysis based on information processing and associated energy consumption (Kaneko and Furusawa, 2018). Nevertheless, the difficulty of measuring the brain's thermodynamic processes, free energy, and entropic properties in neural computation, including our general lack of understanding of emotion and consciousness, may hold this promising field back. Nevertheless, a few pioneering works tackled “*Decision making from the perspective of neural thermodynamics and molecular information processing*.”

As an information transmitter and processor, the brain is part of the environment's thermodynamic cycle through stimuli (Deli and Kisvarday, 2020). The frequencies determine the neuronal information flow and emotional intensity (Kao et al., 2015). Emotions are also involuntary regulators of social interaction, generating competition, and repetitious thinking. Furthermore, cognitive processes demonstrate energy information relationships (Deli and Kisvarday, 2020; Deli et al., 2021). Mental change requires energy, such as mental effort (Bechler et al., 2019; Stringer et al., 2019; Wang et al., 2021) due to a tight coupling between energy-information processing (Esghaei et al., 2022). Therefore, studying the thermodynamic relationships of the brain's intelligent computation is a promising field of study.

Annila's manuscript, “*The fundamental nature of motives*,” examined decision-making using a thermodynamic tenet. He shows how the minimization of free energy achieves the optimization of subjective expected utility. He offers a thermodynamic theory, which may not predict a decision, but can rate decisions by their least-time free energy consumption. Decision-making is a natural process based on resources of structures inherited from the past. From this perspective, cognitive faculty and core decision-making are projections from the past into the future (Deli and Kisvarday, 2020).

The paper “*The mental Maxwell relations: a thermodynamic allegory for higher brain functions*” by O'Neill and Schoth introduces some interesting ideas for future research. Thermodynamics, as well as Maxwell equations, are well-formulated and experimentally verified theories in physics. On the other hand, the theoretical framework in the mental realm is not a rigorous one compared to the abovementioned theories. Moreover, the variables currently used to describe mental phenomena are numerous and imprecise due to the difficulty of finding core variables in thermodynamics and Maxwell relations. However, the authors' attempt to find such physical variables is thought-provoking and worth pursuing.

Entropic-like quantities over time reveal that they can modulate aesthetic decisions by varying degrees of surprise given temporally integrated expectations. Grzywacz and Aleem, in their brilliant paper “*Does amount of information support aesthetic values?*” elaborately describe how the amount of available information may underlie aesthetic values. Such information-based aesthetic values would be significant by competing with others to drive decision-making. They ask, “What is the evidence that the amount of information supports aesthetic values?” Since the measurement of informational volume is entropy, evaluating the contribution of information on aesthetic values, they use Shannon Entropy to probe whether the brain uses entropy or other relevant measures, mainly Fisher information, in aesthetic decisions. Information measures contribute to these decisions: First, the absolute quantity of information can modulate aesthetic preferences for specific sensory patterns. From their view, the amount of information underpins complex aesthetic values, possibly informing the brain on the allocation of resources or the situational appropriateness of some cognitive models.

The paper on “*Functional implications of Dale's law in balanced neuronal network dynamics and decision making*” by Barranca et al. raises some critical issues regarding the validity of Dale's law (define here) for certain neurons and other biological systems. Dale's law plays an important role in decision-making. Their study sheds light on the violation of Dale's law both experimentally and the shared design principle for biological complex computations.

Neacsu et al. investigated “*Synthetic spatial foraging with active inference in a geocaching task*.” Their comprehensive active inference study used Bayesian statistics to maximize model evidence. Foraging is a fundamental skill for survival. Foraging with Bayesian belief is a very realistic approach that reproduces two foraging behavior levels. The Bayesian inference can facilitate a holistic conceptual as well as mechanistic understanding of foraging *via* a geocaching task.

## Author contributions

All authors listed have made a substantial, direct, and intellectual contribution to the work and approved it for publication.

## Conflict of interest

The authors declare that the research was conducted in the absence of any commercial or financial relationships that could be construed as a potential conflict of interest.

## Publisher's note

All claims expressed in this article are solely those of the authors and do not necessarily represent those of their affiliated organizations, or those of the publisher, the editors and the reviewers. Any product that may be evaluated in this article, or claim that may be made by its manufacturer, is not guaranteed or endorsed by the publisher.
